# Human parainfluenza virus type 2 (HPIV2) induced host ADAM8 expression in human salivary adenocarcinoma cell line (HSY) during cell fusion

**DOI:** 10.1186/1471-2180-9-55

**Published:** 2009-03-16

**Authors:** Guo-Feng Ma, Simo Miettinen, Pauliina Porola, Klaus Hedman, Jari Salo, Yrjö T Konttinen

**Affiliations:** 1 Department of Medicine/invärtes medicin, Helsinki University Central Hospital, PO Box 700, FIN-00029 HUS, Helsinki, Finland; 2 Department of Virology, Haartman Institute, PL 21 (Haartmaninkatu 3), FIN-00014 University of Helsinki and Helsinki University Hospital Laboratory Division, Helsinki, Finland; 3 Department of Orthopedics and Traumatology, Helsinki University Central Hospital, PL 22 (Topeliuksenkatu 5), Helsinki, Finland; 4 Department of Medicine/invärtes medicin, Helsinki University Central Hospital, PO Box 700 (Haartamaninkatu 8), FIN-00029 HUS, ORTON Orthopedic Hospital of the ORTON Foundation, Helsinki, Finland; and COXA Hospital for Joint Replacement, Tampere, Finland

## Abstract

**Background:**

The aim of the study was to investigate expression of ADAMs (A Disintegrin and A Metalloproteinase) of host cell origin during cell-cell fusion induced by human parainfluenza virus type 2 (HPIV2).

**Results:**

Induction of host cell ADAM9 was observed in GMK cells, but the applicability of this model was restricted by lack of cross-reactivity of the anti-human ADAM8 antibodies with the corresponding green monkey antigens. HSG cells were not susceptible to HPIV2 virus infection. In contrast, in human parotid gland HSY cells, a natural host cell for paramyxoviruses, HPIV2 induced ADAM8 expression. ADAM8 staining increased dramatically over time from 7.9 ± 3% at zero hours to 99.2 ± 0.8% at 72 hours (p = 0.0001). Without HPIV2 the corresponding percentages were only 7.7% and 8.8%. Moreover, ADAM8 positive cells formed bi- (16.2%) and multinuclear cells (3.5%) on day one and the corresponding percentages on day three were 15.6% for binuclear and 57.2% for multinuclear cells.

**Conclusion:**

ADAM8, well recognized for participation in cell-to-cell fusion especially in osteoclast formation, is up-regulated upon formation of multinuclear giant cells after HPIV2 induction in HSY cells. The virus-HSY cell system provides a novel experimental model for study of the molecular mechanism of cell fusion events.

## Background

In paramyxovirus-host cell fusion the virion membrane and host cell membrane are first brought into close contact and docked to each other. This occurs with the help of the hemagglutinin-neuraminidase on the surface of the virus, which binds to the sialic acid-containing receptor on the surface of the host cell. This interaction triggers the latent fusion protein (F protein) trimers inserted by their carboxy-terminal end into the virion membrane to undergo conformational changes. This exposes their hidden amino-terminal hydrophobic fusion peptide domains. The now exposed hydrophobic viral fusion peptides insert into the host (target) cell membrane. Thus, they anchor the virion to the host target cell. Two close-by anchoring fusion proteins then fold, this time so that their two trimeric membrane-bound hydrophobic domains (i.e. the transmembrane domain fixed in the virion membrane and the fusion peptide domain fixed in the host cell membrane) align in an anti-parallel fashion to form a structurally strong 6-helix bundle. This power stroke brings the virion membrane and the host cell membrane together and leads to exoplasmic virus-host cell fusion followed by formation and expansion of the initial pore between the virus and the host cell. Uncoating of the virus ends up with entrance of the viral RNA and its nucleoproteins into the host cell [1]. Thus, the viral fusion protein helps the viral envelope to fuse directly with the plasma membrane of the target cell [2].

Compared with the understanding of the virus-host cell fusion and entry of the virus into host cell (or an artificial liposome), insight into the molecular mechanisms of the formation of virally induced syncytia (multikaryons) is at a rudimentary level. Fusion of the membranes of the virus-infected cells with those membranes of adjacent uninfected or infected cells results in the formation of a giant virus factory, a syncytium, with the additional advantage from the viral point of view of not destroying the exploited host cell. Some pioneering studies have focused on the lipid, glycoprotein and protein compositions of the target cell membranes and their ability to promote the formation of syncytia [3-5]. Such studies are hampered by the fact that the lipids, glycoproteins and proteins and their receptors on the mammalian cell surfaces of are much more complex than the most elaborate virion membranes and their constituents. We hypothesized that, good fusion molecule candidates of mammalian origin, which could contribute to virally induced host cell-host cell fusion, would be such molecules that have already been recognized in other, non-virally induced cell-cell fusion events.

Fusion of gametes to form the zygote cell requires "A Disintergrin and A Metalloproteinase" molecules known as ADAM1 and ADAM 2 [6,7]; and the myoblast fusion to myotubes requires ADAM12 [8,9]. Macrophage progenitor cell fusion to osteoclasts seems to require ADAM8 [10], ADAM9 and ADAM12 [11]. We have reported that ADAM8 [12], ADAM9 [13] and ADAM12 [14] are involved in the fusion of monocyte/macrophages to foreign body giant cells. Some ADAMs (including ADAM8, ADAM9 and ADAM12) contain a putative fusion peptide in the cysteine-rich domain that is involved in membrane fusion in the formation of multinuclear giant cells and osteoclasts [8-10,15]. A fusion peptide penetrates the lipid bilayer of the cell. Thus, the anchoring fusion peptide propels the cell so close to the target cell membrane that the cell fusion is triggered. We have focused in particular in ADAM8, whereby it was of interest to clarify whether it has the potential to fuse, not only macrophages, but also other cells. Such a system might furthermore provide a novel method for study of cell fusion in general. Thus, ADAM8 was selected as the candidate molecule and was studied for its eventual presence and regulation in virally induced human cell-cell fusion. It is not known whether ADAM8 is regulated or utilized by viruses for spreading their offsprings to uninfected cells and whether this represents an option for the virus to invade additional cells.

Our working hypothesis was that, human parainfuenza virus type 2 (HPIV2), typically forming syncyta, might utilize and/or induce transmembrane ADAM8, a protein linked earlier to the formation of osteoclasts and foreign body giant cells. To test this hypothesis, we added HPIV2 to green monkey kidney (GMK) cells and to examine human salivary gland cell lines (HSG and HSY) to study whether host cell-encoded ADAM8 is involved in the fusion of target cells. The results led to the insight that the HPIV2 induced cell fusion system could provide a novel human cell-based experimental system of study regulation of cell fusion-associated molecules in general.

## Results and Discussion

### ADAMs in HPIV2-infected GMK cells

Green monkey kidney (GMK) cells are in virological laboratories used for maintaining the HPIV2 stocks. Therefore, the effects of HPIV2 on GMK cells were studied first. When these cells were infected by the HPIV2, viral hemagglutinin-neuraminidase antigens were found in infected cells and multinuclear syncytia were formed [16]. In these preliminary experiments, the eventual involvement of ADAMs was studied by using affinity purified polyclonal rabbit anti-human ADAM8 antibodies. The human specific ADAM8 antibody did not show cross-reactivity with the corresponding green monkey kidney cell (although positive sample controls stained in parallel with the GMK cells were positive), whereby ADAM8 could not be assessed.

**Figure 1:**
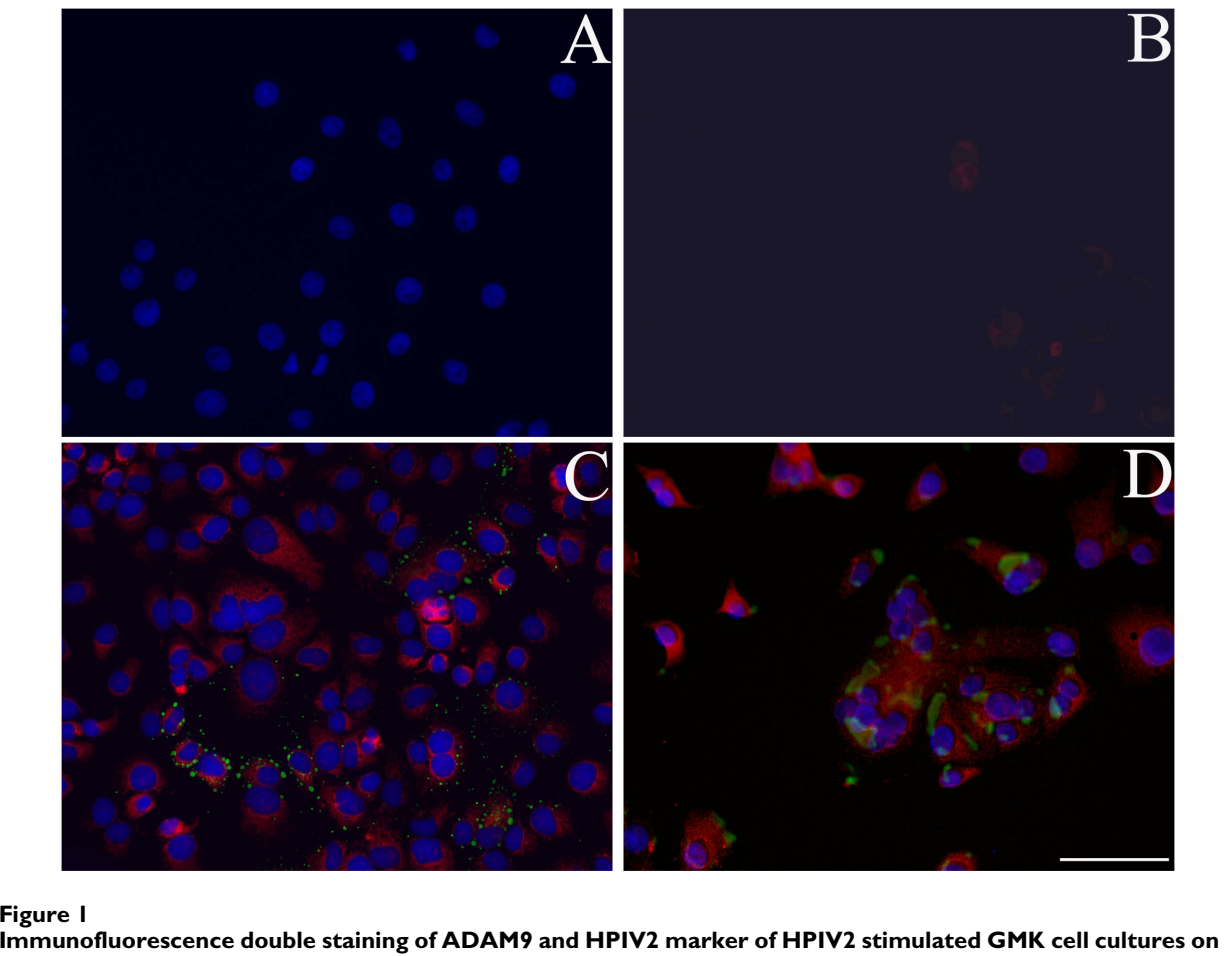
**Immunofluorescence double staining of ADAM9 and HPIV2 marker of HPIV2 stimulated GMK cell cultures on culture day 0 (panel A, B), 1 (panel C), 3 (panel D)**. ADAM9 staining is shown in red and HPIV2 shown in green, together with the blue nuclear counterstain of the same field. On culture day 0 HPIV2 did not infect any cells, therefore (A) shows only nuclear counterstain, (B) shows only ADAM9 staining, (C, D) overlays show double staining of ADAM9 and HPIV2 marker on culture days 1 and 3, respectively. Bar = 10 μm.

At 2 hours HPIV2 antigens were not yet found in infected GMK cells (data not shown) and ADAM9 was absent (Figure 1A, B). On culture day 1 HPIV2 was seen in infected GMK cells and all the infected and some of the uninfected GMK cells were ADAM9 positive (Figure 1C). On culture day 3 HPIV2 had infected most GMK cells and had caused cytopathic effects including formation of large multinucleated syncytia. The multinuclear giant cells were relatively strongly labeled for ADAM9 (Figure 1D). The positive controls of ADAMs were positive showing that the immunolabeling protocol used worked acceptably; also the negative staining controls were negative showing that the ADAM9 staining results were correctly positive (data not shown).

This is in line with our previous study demonstrating that human ADAM9 might as a human protein participate in the formation of multinuclear osteoclasts and foreign body giant cells [13]. However, due to the technical limitations of the HPIV2-GMK system (cross-species differences in the ADAM8 antigen), it was decided that further attempts be done using target cells of human origin.

### ADAM8 expression in the HPIV2 infected HSY cells

HPIV2 infection of GMK cells gave promising results but ADAM8, our main target of interest, could not be shown in these monkey cells using anti-human antibodies. Human submandibular cell line HSG was then used, but it was not possible to infect HSG cells with HPIV2. No hemagglutinin-neuraminidase antigens were found in HSG cells in co-cultures with HPIV2 virus and no syncytia were formed. As HPIV2 is a paramyxovirus, and the virus causing mumps (human epidemic parotitis) with clear preference to human parotid glands, next a human parotid gland cell line HSY was tried.

**Figure 2:**
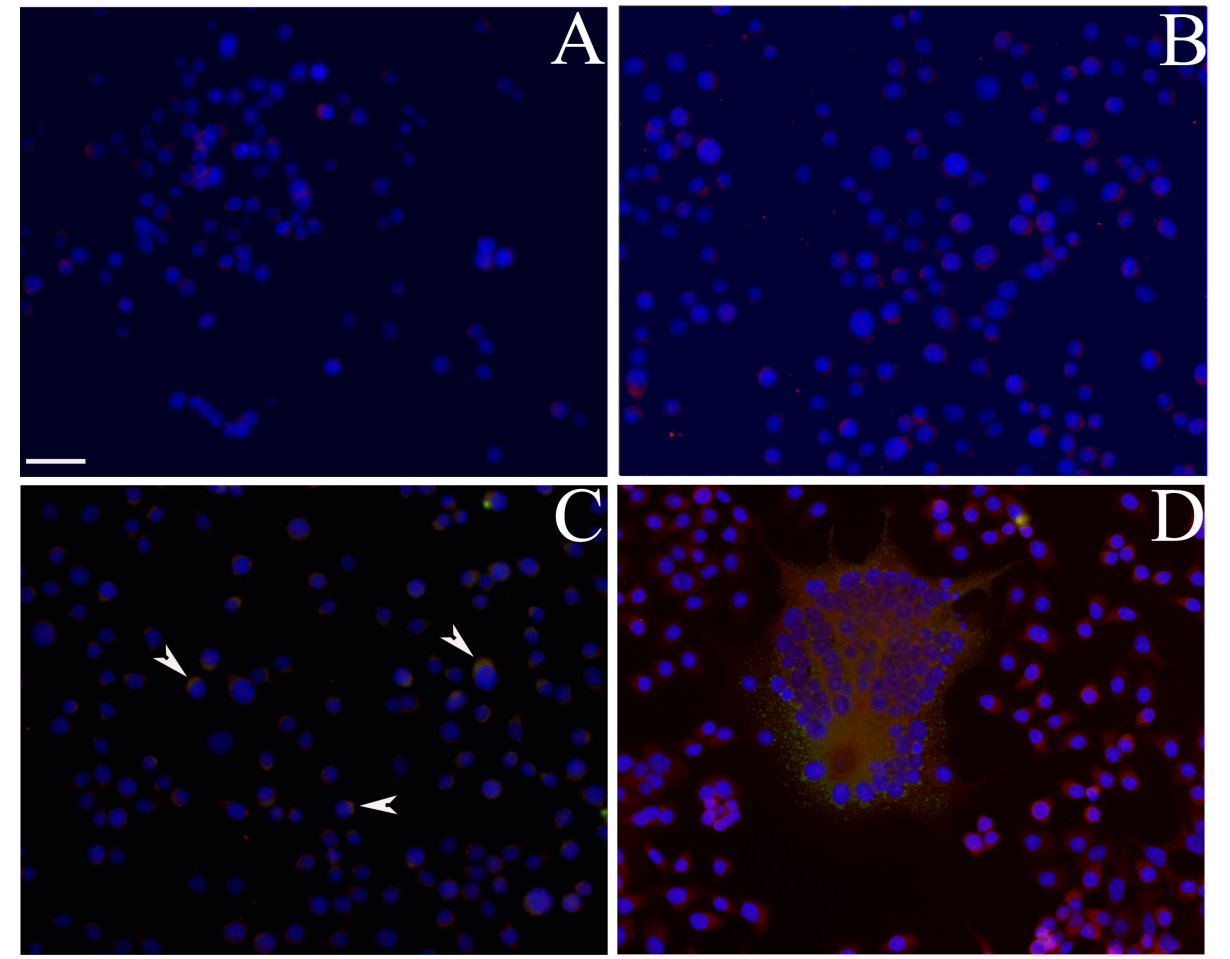
**Immunofluorescence double staining of ADAM8 and HPIV2 marker of HPIV2 stimulated HSY cell cultures on culture day 0 (panel B), 1 (panel C), 3 (panel D)**. ADAM8 staining is shown in red and HPIV2 shown in green (arrowheads), together with the blue nuclear counterstain of the same field. Panel A shows the staining of HSY cells that HPIV2 did not infect as negative control, therefore (A) only ADAM8 weak staining with nuclear counterstain, (B, C, D) overlay of double staining of ADAM8 and HPIV2 marker with blue nuclear counterstain of the same field on culture days 0, 1 and 3, respectively. Bar = 10 μm.

**Figure 3:**
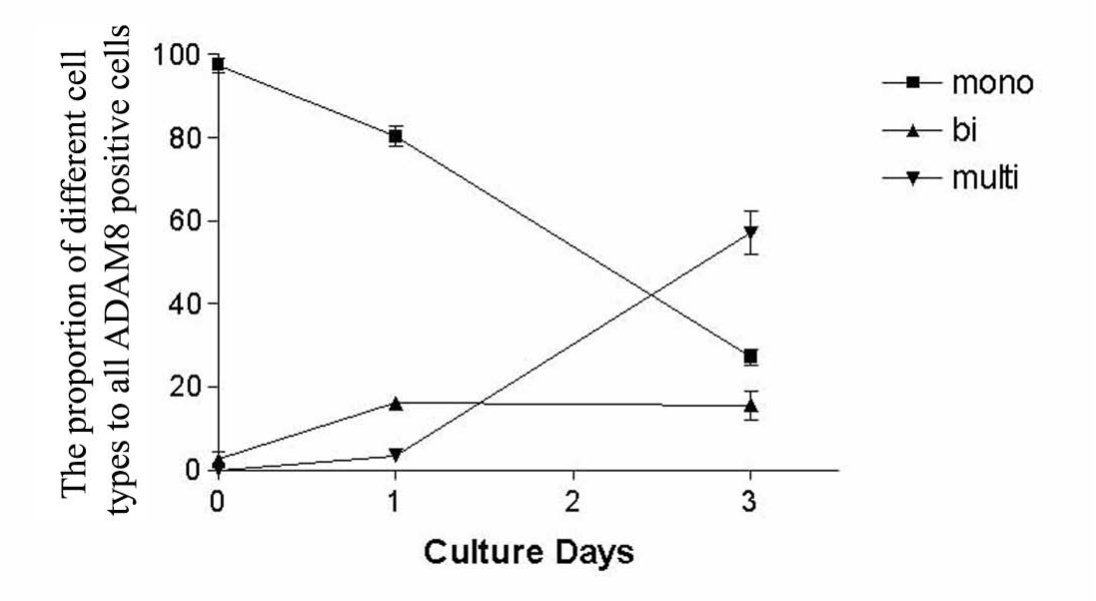
**The proportion of mononuclear (black square), binuclear (black upwards pointing triangle) and multinuclear positive cells (downwards pointing triangle) of all ADAM8 positive cells in the immunofluorescence staining of ADAM8 in HPIV2 stimulated human salivary adenocarcinoma cell cultures on culture days 0, 1 and 3 as a function of time**.

In the uninfected HSY cells a very weak ADAM8 signal was seen (Figure 2A). At 2 hours HPIV2 was not yet found in HPIV2 infected HSY cell cultures and ADAM8 showed weak staining (Figure 2B). On culture day one, HPIV2 was seen inside HSY cells, which usually also showed cytoplasmic patches of immunoreactive ADAM8 (Figure 2C). On culture day three HPIV2 was found in some HSY cells. In addition, many large multinucleated cells were seen, which also were HPIV2 positive. In double label studies they stained for ADAM8, with a relatively strong signal, and a non-homogenous, granular and patchy cytoplasmic distribution (Figure 2D). In morphometric analysis, without HPIV2 stimulation the percentage of ADAM8 positive cells at 2 hours was 7.7 ± 0.9%, at 24 hours 7.5 ± 0.9% and at 72 hours 8.8 ± 1.0%. In HPIV2 infected cultures of human HSY cells the percentage of ADAM8 positive cells at 0 hour was 7.9 ± 3%, at 2 hours 15.0 ± 6.7% (p = 0.25), at 24 hours 57.0 ± 11% (p = 0.0719) and at 72 hours 99.2 ± 0.8% (p = 0.0001). All HPIV2 infected cells were also ADAM8 positive. We then calculated the percentages of ADAM8 and HPIV2 double positive cells and obtained that way also the number of ADAM8 positive but HPIV2 negative cells (Table 1). Moreover, ADAM8 positive cells formed also bi- and multinuclear cells. Fusion was seen already on day one at which time 16.2 ± 1.0% of the cells were binuclear and 3.5 ± 0.8% were multinuclear (all of them being ADAM8 positive). On day 3 15.6 ± 2.5% of the cells were binuclear (and all of them also ADAM8 positive) and altogether 57.2 ± 3.8% of all cells were multinuclear (and all of the also ADAM8 positive) (Figure 3).

**Table 1:** Percentage of positive cell of all the cells (mean ± SEM %)

	Day 0	Day 1	Day 3
Percentage of ADAM8+/HPIV2+ cells	0	19.1 ± 2.9	19.3 ± 1.5

Percentage of ADAM8+/HPIV2- cells	15 ± 6.7	37.9 ± 3.6	78.9 ± 1.9

Expression of ADAM8 HPIV2 infected cell cultures was studied using the rabbit anti-human ADAM8 carboxy-terminal antibody as it was reasoned that the antibody recognizing the intracytoplasmic carboxy-terminal end of the molecule would provide an idea of the amount of the full-length ADAM8 molecule, with the amino-terminal propeptide and metalloproteinase domains, as well as its amino-terminal end trimmed counterparts. Indeed, in non-infected HSY cells the proportion of ADAM8-positive cells was relatively low and stable over time. In contrast, HPIV2 clearly and dramatically up-regulated ADAM8 expression, which in only 3 days increased from 7.9 to 99.2% (p < 0.001). Apart from this dramatic up-regulation of host cell encoded ADAM8, two other interesting observations were made in these experiments. First, this increase in ADAM8 expression was accompanied by the formation of binuclear cells and very soon also of multinuclear syncytia. By kinetic association between the increased ADAM8 expression and cell-to-cell fusion it was concluded to indicate that HPIV2 induces this tentative host fusion molecule for enhancement of host-host cell fusion. This conclusion is in part based on the general role of ADAM8 in such fusion processes in the formation of osteoclasts [10] and foreign body giant cells [12]. It can also be asked whether this host-host cell fusion could provide some survival advantages to the HPIV2 virus. Interestingly, it was noticed that at the beginning of the culture period most of the ADAM8-positive host cells were negative for HPIV2 hemagglutinin-neuraminidase antigen indicating that they were non-infected. However, it is also conceivable that the detection of nucleocapsid protein, the most abundant viral protein, would have raised the number of cells identified as HPIV2-positive. Cells, which were not immunoreactive for HPIV2, were often seen in close association to the HPIV2-immunoreactive mononuclear, binuclear and multinuclear cells apparently ready to undergo fusion with their infected partners. The infection of host cells by HPIV2 triggers some unknown mechanisms which initiate cell fusion process and these mechanisms seem to lead to up-regulation of host cell ADAM8, which might contribute to the cytopathic cell fusion. This suggests that HPIV2 utilizes host encoded ADAM8 to spread from infected to non-infected target cells. On the cell surface, host cell fusion molecules, like ADAMs, could cause the HPIV2 infected host cell membrane to fuse with the neighboring non-infected cells to form syncytia. This strategy might enable fusion of dozens of non-infected cells to a giant multi-nuclear cell which means that HPIV2 can use resources of many more cells compared to an infection of only one cell although "syncytial" infected cells will lose viability much faster than do "non-syncytial" infected cells. At the same time, this syncytial virus factory protects against host-derived anti-viral antibodies, complement and other host defense factors, unable to penetrate to the host target cell cytoplasm upon virus reproduction. However, expression of an ADAM8 protein in mononuclear prefusion cells and multinucleated cells does not mean that it functions as a fusion protein in this context although there is evidence for this in human osteoclastogenesis [17].

## Conclusion

This study demonstrates for the first time the up-regulation of ADAM8 during HPIV2 induced cell fusion. Using a Trojan horse strategy of this kind HPIV2 can spread efficiently and safely, possibly in part by utilizing the fusion molecules of the host cells. Mammalian cell fusion has been studied by others and by us in human monocyte cultures stimulated with receptor activator of nuclear factor kappa B ligand, which however is quite a time consuming and complicated system [18,19]. It was therefore the aim of the present work to assess if HPIV2 infected human cells have a potential to utilize also host cell fusion molecules in the fusion process as the first step towards the development of a novel tool for studying fusion of human cells although the characteristics of this system were not clarified by this work.

## Methods

### Cell cultures

GMK, a kidney-derived epithelial-like cell line, is susceptible to HPIV2 and was maintained in virological laboratories to generate HPIV2 virions. It was obtained from the Helsinki University Central Hospital laboratory and maintained in minimal essential medium (MEM, HaartBio Ltd. Helsinki, Finland) containing 10% (v/v) heat-inactivated foetal bovine serum and 100 μg/l Glutamine-Penicillin-Streptomycin (HaartBio) in 75 cm^2 ^culture flasks at 37°C and 5% CO_2 _incubator [20].

HSG cell line derived from human submandibular gland [21] and HSY cell line derived from human parotid gland [22] were cultured at 37°C, 5% CO_2_-in-air in Dulbecco's modified Eagle's medium with nutrient mixture F-12 Ham (DMEM/F-12, Sigma, St. Louis, MO) supplemented with 10% (v/v) fetal calf serum, 2.2 mM glutamine, 100 U/ml penicillin and 100 μg/ml streptomycin.

### Virus infection

HPIV2 was obtained from the Helsinki University Central Hospital laboratory where it was used as a reference virus. The virus was grown in the GMK cells according to standard procedures [23].

Medium was removed when the adherent GMK, HSG or HSY target cells covered 70–90% of the surface in 75 cm^2 ^cell culture flasks. They were washed with 1 ml culture medium twice and then exposed to 1.5 μg/ml trypsin at +37°C and in 5% CO_2 _for 5 minutes. The detached GMK, HSG, HSY cells were divided into the 6-well plates, which 1.2 × 10^6 ^cells per well. 23 μl HPIV2 primary viral suspension, containing 2.7 × 10^7 ^infective units per milliliter, was added to each 2.5 ml cell culture well. The cells were harvested at zero hours (before addition of HPIV2), at two hours, on day one and on day three. In parallel, cells cultured without HPIV2 were harvested as controls at zero and two hours and on one and three days.

### Double-immunofluorescence staining

GMK cells cultured on coverslips for 2 hours, 1 day or 3 days were washed in 10 mM phosphate buffered, 150 mM saline, pH 7.4 (PBS), fixed in pure acetone for 20 minutes at -20°C and incubated in 1) a mixture of 2 μg/ml polyclonal rabbit anti-human ADAM9 IgG (Triple Point Biologics, Forest Grove, OR) and fluorescein isothiocyanate (FITC) labeled monoclonal mouse anti-HPIV2 hemagglunin-neuraminidase IgG_1 _(Light Diagnostic Respiratory DFA Viral Screening & Identification Kit, Millipore, Temecula, CA, USA) for 30 minutes and 2) Alexa Fluor 594-labeled goat anti-rabbit IgG (Molecular Probes, Eugene, OR) for 30 minutes. Non-immune rabbit IgG and monoclonal mouse IgG_1 _of irrelevant specificity were used at the same concentration instead of the primary antibodies as negative controls. Synovial membrane-like interface tissue samples collected from the proximal bone-cement or bone-stem interfaces around aseptically loosened femoral stems during revision total hip replacement operations were used as positive controls [13]. Coverslips were mounted using Vectashield Mounting Medium containing 4', 6-diamidino-2-phenylindole (DAPI, Vector Laboratories, Burlingame, CA) for nuclear staining.

HSY cells cultured on coverslips for 2 hours, one day or three days were washed in PBS, fixed in pure acetone for 20 minutes at -20°C and incubated in 1) a mixture of affinity purified rabbit anti-human (carboxy-terminal end of, proprietary information) ADAM8 IgG (Cedarlane Laboratories Ltd., Hornby, Ontario, Canada) and FITC-labeled monoclonal mouse anti-HPIV2 IgG_1 _(Millipore) for 30 minutes and 2) Alexa Fluor 594-labeled goat anti-rabbit IgG (Molecular Probes) for 30 minutes. The antibody used for ADAM8 staining has been peptide-affinity purified and does not react with other ADAMs. Coverslips were mounted using Vectashield Mounting Medium containing DAPI (Vector Laboratories). Non-immune rabbit IgG and monoclonal mouse IgG_1 _of irrelevant specificity were used at the same concentration instead of the primary antibodies as negative staining controls. Stained cells were observed using Olympus motorized revolving AX 70 system microscope (Olympus Optical, Hamburg, Germany) coupled with 12 bits Sensicam digital image camera (Sensicam, Kelheim, Germany) and analyzed using the Analysis Pro 3.0 image analysis and processing system (Soft-Imaging Software GmbH, Munster, Germany).

## Abbreviations

ADAM: A Disintegrin and A Metalloproteinase; DAPI: 4', 6-diamidino-2-phenylindole; FITC: fluorescein isothiocyanate; GMK: green monkey kidney cell line; HSG: human salivary gland cell line; HSY: human salivary adenocarcinoma cell line; MEM: minimal essential medium; PBS: phosphate buffered saline; HPIV2: human parainfluenza virus type 2

## Authors' contributions

GFM carried out viral and cell cultures, immunofluorescent staining and wrote the manuscript. SM cultured the GMK cells. PP cultured the HSG and HSY cells. KH provided the lab facilities, and participated in writing. JS participated in the design and coordination. YTK participated in its design and coordination and help to draft the manuscript. All authors read and approved the final manuscript.
